# Supplementary material for: Competition, capital growth and risk-taking in emerging markets: Policy implications for banking sector stability during COVID-19 pandemic
Source: PLoS One. 2021 Jun 24;16(6):e0253803. doi: 10.1371/journal.pone.0253803 (PMC8224939; doi:10.1371/journal.pone.0253803)
Supplement: S1 Appendix — (DOCX) [file pone.0253803.s001.docx]

**S1 Appendix**

**S1 Table. Dependent and explanatory variables**

| **Variable** | **Explanation** | **Data Source** |
| --- | --- | --- |
| **Dependent Variables** |  |  |
| ***Capital ratios*** |  |  |
| Eligible Capital/Total Assets (EC/TA) | Ratio of total eligible capital to total assets | Authors’ calculations based on Orbis Bank Focus |
| Total Equity/Total Assets  (TE/TA) | Ratio of total equity to total assets | Authors’ calculations based on Orbis Bank Focus |
| **Independent Variables** |  |  |
| ***Credit risk measures*** |  |  |
| Loan loss reserves/ Gross loans (LLR/GL) | Bank reserves for loan loss divided by gross loans times 100 | Authors’ calculations based on Orbis Bank Focus |
| Non-performing loans/ Gross loans (NPL/GL) | Bank non-performing loans divided by gross loans times 100 | Authors’ calculations based on Orbis Bank Focus |
| ***Market Competitiveness*** |  |  |
| HH-Index | Herfindahl-Hirschman index (HHI) is a measure of market concentration calculated as the sum of squared banks’ market shares. | Authors’ calculations based on Orbis Bank Focus |
| H-Statistic | The H-Statistic is estimated using cross-sectional regressions with total revenue for each country during the period 2006-2018 | Authors’ calculations based on Orbis Bank Focus |
| Lerner index | The index is measured by the difference between the output price of a firm and the marginal cost divided by the output price. The value of the index varies between 0 and 1, such a higher Lerner value (i.e. close to 1) indicate monopoly situation. | Authors’ calculations based on Orbis Bank Focus |
| ***Regulation and institutions*** |  |  |
| Activity Restrictions | The indicator is constructed as an index and takes on values between (1) and (4), whereby the activities are classified as unrestricted (1), permitted (2), restricted (3), or prohibited (4), with possible index variation between four and sixteen. Higher values indicate greater restrictions on bank activities and non-financial ownership and control | The World Bank's bank regulation survey (Survey IV, 2012) |
| Institution | The index of institution is the average of six indicators: 1) voice and accountability, (2) political stability and absence of violence, (3) government effectiveness, (4) regulatory quality, (5) rule of law, and (6) control of corruption. | [64] The World Bank, WGI 2004-2018;  [92] Kaufmann et al. (2008) |
| ***Ownership Indicators*** |  |  |
| Ownership concentration | The level of ultimate ownership held by the largest shareholder | Orbis Bank Focus and annual reports (2006-2018) |
| Government ownership | Proportion of equity held by the government | Orbis Bank Focus and annual reports (2006-2018) |
| Foreign ownership | Proportion of equity held by foreign investors | Orbis Bank Focus and annual reports (2006-2018) |
| ***Bank-level Characteristics*** |  |  |
| Return on Asset (ROA) | Ratio of pre-tax income to total assets | Orbis Bank Focus and authors’ calculation |
| Cost-to-income ratio (CIR) | The share of bank operating costs to bank operating income before provisions times 100 | Orbis Bank Focus and authors’ calculation |
| Deposit/ Total Assets | Bank characteristic calculated as deposits divided by total assets | Orbis Bank Focus and authors’ calculation |
| Loan/Total Assets | Bank characteristic calculated as loans over total assets | Orbis Bank Focus and authors’ calculation |
| Loan/Total Deposits | Bank characteristic calculated as loans over total deposits |  |
| Revenue Diversification | Ratio of non-interest revenue to total assets | Orbis Bank Focus and authors’ calculation |
| Leverage | Ratio of equity capital to total liability | Orbis Bank Focus and authors’ calculation |
| Size | Log of a bank's total assets | Orbis Bank Focus and authors’ calculation |
| ***Macroeconomic Variables*** |  |  |
| GDP Growth | Annual percentage growth rate of GDP (constant 2005 U.S. dollars), proxy for market size | [65] The World Bank Development Indicators, 2006-2018 |
| Inflation | Inflation is measured by the consumer price index, in percent | [65] The World Bank Development Indicators, 2006-2018 |
