# Supplementary material for: Competition, capital growth and risk-taking in emerging markets: Policy implications for banking sector stability during COVID-19 pandemic
Source: PLoS One. 2021 Jun 24;16(6):e0253803. doi: 10.1371/journal.pone.0253803 (PMC8224939; doi:10.1371/journal.pone.0253803)
Supplement: S2 Appendix — (DOCX) [file pone.0253803.s002.docx]

**S2 Appendix**

**The Herfindahl-Hirschman (HHI) index**

The Herfindahl-Hirschman index (HHI) is an alternative measure of market concentration often used by various scholars [83]. The sum of square of the percentage of market share (using total deposits) of all the banks in a given country and given period gives the HHI index. This measure is more data intensive than the concentration ratio, as it requires data about the entire bank size distribution of the participant banks in the industry. It means it uses the market share of each bank in the banking industry. The model for estimating HHI index is specified as follows:

$HHI= \sum_{1}^{N} S_{i}^{2}$ , (S2.1)

where 𝑁 is the total number of banks in the market, 𝑠_𝑖_ is the market share of the 𝑖_𝑡ℎ_ bank.

The HHI index is a positive figure, ranging between close to zero for perfectly competitive banking market to 1 for a monopoly where a single bank controls all the bank market, that is, (0 < HHI ≤ 1). The lower the HHI index is, the more competitive the banking market is. The extent of concentration of the banking industry based on the index is given in the table (see Table S2).

S2 Table. HH-Index and degree of concentration [83]

| **HH-Index** | **Degree of Competition/Concentration** |
| --- | --- |
| Less than 0.15 | Competitive (unconcentrated) markets |
| Between 0.15 and 0.25 | Moderately concentrated markets |
| Greater than 0.25 | Highly concentrated markets |

**The H-Statistic**

This study uses Panzar-Rosse (PR) model which is an econometric approach in which competitive market conditions are to be assessed quantitatively. The model determines the competitiveness behavior of banks as per the comparative static features based on a reduced form of revenue equations using cross-section data [72, 71, 78] indicated that summing elasticity of the reduced form of revenues gives the so-called H-statistic, on which the model is based. The H-statistics ranges from negative infinity (-∞) to +1. The greater the value of H-stat the greater the competition is, a value of +1 indicating perfect competition [79, 80], in which total revenue and input prices increase by the same percentage. In the model it is assumed that banks have cost and revenue functions which define profit maximization path, in which marginal cost should be equal to the marginal revenue. To approximate the H-statistic empirically, we use a set up similar to [8, 72, 73]:

$LnTRit = + \beta1Ln(P1it) + \beta2Ln(P2it) + \beta3Ln(P3it) + \cdots\beta nLn(Pnit) + \beta Conit + ℇit$ *,*

(S2.2)

where

- the superscript *i* denotes bank *i*, and the uperscript *t* denotes year *t*;
- 𝑇𝑅_𝑖𝑡_ – interest revenue to total assets (proxy for output price);
- 𝑃_1𝑖𝑡_ – is the ratio of interest expenses to total deposits and money market funding (proxy for input price of deposits);
- 𝑃_2𝑖𝑡_ – is the ratio of personnel expenses to total assets (proxy for input price of labor);
- 𝑃_3𝑖𝑡_ – is the ratio of other operating and administrative expenses to total assets (proxy for input price of equipment/fixed capital);
- 𝐶𝑜𝑛_𝑖𝑡_ – is a matrix of controls including the ratio of equity to total assets, the ratio of net loans to total assets, and the logarithm of assets (to control for bank size effects);
- ε_it_ – random error.

To obtain value for the H-Statistic for each year, it is common in the literature to estimate a reduced-form model whereby output is regressed on factor input prices and some controls that shift the bank’s revenue function [14]. The H-Statistic equals the sum of the input price elasticities of total revenues:

$H-Statistic = \sum(\beta1 + \beta2 + \beta3)$ (S2.3)

The magnitude of H-Statistic can be interpreted in the following way:

- H = 1, indicates perfect competition.
- 0 < H > 1, indicates monopolistic competition.
- H ≤ 0, indicates monopoly equilibrium, perfectly colluding oligopoly or conjectural variations short- run oligopoly.
