# Supplementary material for: Competition, capital growth and risk-taking in emerging markets: Policy implications for banking sector stability during COVID-19 pandemic
Source: PLoS One. 2021 Jun 24;16(6):e0253803. doi: 10.1371/journal.pone.0253803 (PMC8224939; doi:10.1371/journal.pone.0253803)
Supplement: S3 Appendix — (DOCX) [file pone.0253803.s003.docx]

**S3 Appendix**

**S3 Table. Determinants of bank competition (H-Statistic)**

| **Explanatory Variables** | **Dependent variable: H-Statistic** | | | |
| --- | --- | --- | --- | --- |
|  | **Model 1** | **Model 2** | **Model 3** | **Model 5** |
| Constant | -0.460 | -0.357 | -0.466 | -0.300 |
|  | (0.115) | (0.210) | (0.114) | (0.280) |
| Official | 0.008*** |  |  |  |
|  | (0.000) |  |  |  |
| Capital |  | 0.012*** |  |  |
|  |  | (0.000) |  |  |
| Restrict |  |  | 0.009*** |  |
|  |  |  | (0.000) |  |
| Private Monitoring |  |  |  | 0.007*** |
|  |  |  |  | (0.000) |
| Loan Loss Reserves/Gross Loans | -0.101* | -0.105* | -0.092* | -0.107** |
|  | (0.066) | (0.052) | (0.088) | (0.049) |
| Pre-tax ROA | 0.234 | 0.226 | 0.251 | 0.208 |
|  | (0.486) | (0.496) | (0.455) | (0.528) |
| Cost-income ratio | 0.008*** | 0.008*** | 0.008*** | 0.008** |
|  | (0.000) | (0.000) | (0.000) | (0.025) |
| Log(Total Assets) | 0.003** | 0.003** | 0.003** | 0.003** |
|  | (0.015) | (0.013) | (0.016) | (0.024) |
| Institutional Quality Index | -0.146*** | -0.152*** | -0.145*** | -0.150*** |
|  | (0.000) | (0.000) | (0.000) | (0.000) |
| GDP per capita | 0.270*** | 0.242*** | 0.272*** | 0.227*** |
|  | (0.000) | (0.001) | (0.000) | (0.001) |
| GDP Growth | -1.049*** | -0.993*** | -1.101*** | -0.990*** |
|  | (0.000) | (0.000) | (0.000) | (0.000) |
| Inflation | 0.249*** | 0.224** | 0.240*** | 0.212** |
|  | (0.0005) | (0.011) | (0.007) | (0.014) |
| ISLAMIC_D | 0.001 | 0.001 | 0.001 | 0.001 |
|  | (0.871) | (0.869) | (0.860) | (0.877) |
| Country Dummy | Yes | Yes | Yes | Yes |
| Year Dummy | Yes | Yes | Yes | Yes |
| Number of Observations | 2901 | 2901 | 2901 | 2901 |
| Adj. R-squared | 0.5483 | 0.5462 | 0.5506 | 0.5436 |

The table shows the empirical results from OLS estimation with heteroskedasticity-robust standard errors. The H-Statistics is dependent variable computed in S2 Appendix. Robust *p*-statistics are shown in parentheses. *, **, and *** denote significance at the 10, 5 and 1 percent, respectively
